# Automatic Identification of Referral-Warranted Diabetic Retinopathy Using Deep Learning on Mobile Phone Images

**DOI:** 10.1167/tvst.9.2.60

**Published:** 2020-12-04

**Authors:** Cassie A. Ludwig, Chandrashan Perera, David Myung, Margaret A. Greven, Stephen J. Smith, Robert T. Chang, Theodore Leng

**Affiliations:** 1 Department of Ophthalmology, Byers Eye Institute at Stanford, Stanford University School of Medicine, Palo Alto, CA, USA; 2 Retina Service, Department of Ophthalmology, Massachusetts Eye and Ear, Harvard Medical School, Boston, MA 02114, USA; 3 Department of Chemical Engineering, Stanford University, Stanford, CA, USA; 4 Department of Ophthalmology, Wake Forest Baptist Health, Winston Salem, NC, USA; 5 VA Palo Alto Health Care System, Palo Alto, CA, USA

**Keywords:** artificial intelligence, deep learning, diabetic retinopathy, mobile technology, fundus photography

## Abstract

**Purpose:**

To evaluate the performance of a deep learning algorithm in the detection of referral-warranted diabetic retinopathy (RDR) on low-resolution fundus images acquired with a smartphone and indirect ophthalmoscope lens adapter.

**Methods:**

An automated deep learning algorithm trained on 92,364 traditional fundus camera images was tested on a dataset of smartphone fundus images from 103 eyes acquired from two previously published studies. Images were extracted from live video screenshots from fundus examinations using a commercially available lens adapter and exported as a screenshot from live video clips filmed at 1080p resolution. Each image was graded twice by a board-certified ophthalmologist and compared to the output of the algorithm, which classified each image as having RDR (moderate nonproliferative DR or worse) or no RDR.

**Results:**

In spite of the presence of multiple artifacts (lens glare, lens particulates/smudging, user hands over the objective lens) and low-resolution images achieved by users of various levels of medical training, the algorithm achieved a 0.89 (95% confidence interval [CI] 0.83–0.95) area under the curve with an 89% sensitivity (95% CI 81%–100%) and 83% specificity (95% CI 77%–89%) for detecting RDR on mobile phone acquired fundus photos.

**Conclusions:**

The fully data-driven artificial intelligence-based grading algorithm herein can be used to screen fundus photos taken from mobile devices and identify with high reliability which cases should be referred to an ophthalmologist for further evaluation and treatment.

**Translational Relevance:**

The implementation of this algorithm on a global basis could drastically reduce the rate of vision loss attributed to DR.

## Introduction

The rising prevalence of diabetes mellitus globally, particularly within resource-limited low- and middle-income countries, is of great concern.^1^^,^^2^ There were an estimated 451 million people living with diabetes in 2017 with an estimated expenditure of approximately $850 billion USD.^3^ The prevalence is expected to increase to 693 million by 2045.^3^ With the increasing prevalence of diabetes and increasing life-expectancy of diabetics, the prevalence of diabetic retinopathy (DR) is also expected to rise to 191 million individuals by 2030.^4^ DR is a serious threat to the quality of life of diabetics, accounting for approximately 2.6% of blindness worldwide in 2010.^5^ This surge in prevalence will continue to incite a need for greater and greater numbers of DR screening examinations, increasing visits to optometrists and ophthalmologists alike.

Screening is critical to prevent vision loss caused by DR. Unfortunately, many at risk for diabetic retinopathy do not undergo regular screening either because of poor infrastructure for detection, limited availability of eye care specialists, or cost.^6^^,^^7^ This poor access to health care may result in vision loss as the presenting sign of diabetes mellitus.

In an attempt to address the increasing volume of patients requiring screening, researchers have turned to artificial intelligence (AI) and more specifically deep learning (DL) with convolutional neural networks to automate the diagnosis of referral-warranted diabetic retinopathy (RDR). ^8^^,^^9^ DL is an AI technique that learns through training from large volumes of data. It has been used recently in identifying risk factors for cardiovascular disease within fundus photographs including age, sex, smoking, and systolic blood pressure.^10^ It is being used in electronic health records to predict patient outcomes, determine new risk factors, and assist in documentation.^11^ In some circumstances, AI algorithms have been shown to outperform humans in diagnostic tasks.^12^ Although AI addresses the screening bottleneck, the issue of access to care—both due to geographic restriction and to cost—remains.

With improved geographic availability and reduced cost, smartphone (SP) cameras have emerged as a potential solution. Growing even faster than diabetes is the rise of SP usage, with 94%, 77%, 68%, 30%, and 22% of people owning SPs in South Korea, the United States, China, Kenya, and India, respectively.^13^ These numbers are expected to continue rising. SPs provide the combination of high-resolution cameras, powerful computer processing, and global positioning systems that can allow for fast image capture, diagnosis, and localization to connect patients in need to providers.

Recent advances in SP camera resolution, adapters for fundus imaging, and DL algorithms have made the transition from an SP captured image to diagnosis feasible. To evaluate this technology, we assessed the performance of a DL algorithm in the detection of RDR on low-resolution fundus images acquired with a SP and indirect ophthalmoscope lens adapter.

## Methods

### Fundus Image Data Set

**Figure 1.**
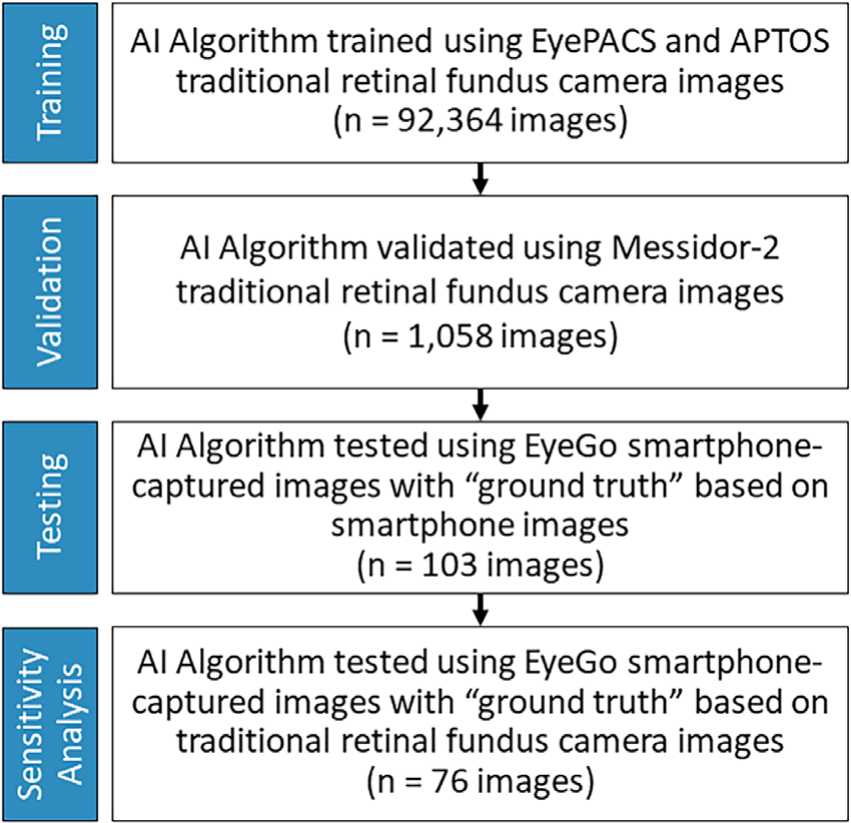
Flow diagram for algorithm training, validation, testing, and sensitivity analysis.

Research adhered to the tenets of the Declaration of Helsinki. We derived our algorithm from a data set of 92,364 color fundus images obtained from the EyePACS public data set (Eye-PACS LLC, Berkeley, CA, USA) and the Asia Pacific Tele-Ophthalmology Society 2019 blindness detection dataset (Fig. 1). These data sets are heterogeneous, containing images from patients across the spectrum of demographic features and that were obtained with varying camera models from many clinical settings.^14^^,^^15^ Images were associated with a label of 0 or 1 referring to no RDR or RDR, respectively, as determined by a panel of medical specialists. In total, 73,723 images were labeled as no RDR whereas 18,641 were labeled as RDR.

### Model Selection

Our model featured deep learning methods to automate characterization of fundus photography. Specifically, deep convolutional neural networks (CNNs) use convolutional parameter layers to learn iteratively how to transform input images into hierarchical feature maps. A CNN architecture was chosen because they tend to have the best performance in image recognition tasks, as seen in the ImageNet challenge.^16^ Given the large number of images available for training, the decision was made to choose a relatively large CNN to help maximize the available data. We chose the DenseNet 201 architecture that has 201 layers, 20 million parameters, and a history of exceptional performance in image recognition tasks.^17^ The model was initialized using the pretrained weights from ImageNet. This allows the model to use the benefits of transfer learning whereby the model has already learnt to recognize basic features of images.

### Data Augmentation

We used data set augmentation methods to encode invariances in the deep learning procedure. To increase image heterogeneity, we encoded rotational, zoom, contrast, and brightness invariance, as well as perspective warping into the data augmentation scheme.

### Artificial Intelligence Training Process

The Fast. AI library (which uses PyTorch) was used for the creation of the AI model used in this study. Analysis was performed using an Intel Xeon CPU @ 2.2Ghz, 13GB RAM, and a Tesla P100 (16GB VRAM) graphics processing unit (GPU). Images were first preprocessed as described above, then the 92,364 image training dataset was split into training (80%) and validation (20%) datasets. Data augmentations were applied on the fly as the model was training. A one-cycle policy was used to maximize the speed of training.^18^ The DenseNet model was first split into the convolutional layers (i.e., the backbone of the model) that contained the pretrained ImageNet weights and the head—a combination of the last few linear layers of the model together with a new prediction layer. For the first four epochs of training, the backbone was kept frozen, with only the head trained. Then the learning rate was optimized (using a learning rate from 3 × 10^−^^6^ to 3 × 10^−^^4^), the backbone of the model was unfrozen, and a further four epochs of training were conducted until convergence. In total, the model took two hours and 20 minutes to train.

### External Validation

We externally validated our algorithm with an independent data set: Messidor-2 (1058 images from four French eye institutions; 675 no RDR, 383 RDR).^19^ The model was only exposed to the training dataset prior—neither the EyeGo nor the Messidor-2 images were used for training.

### Model Testing on EyeGo Dataset

We then tested this DR deep learning algorithm on a dataset of usable fundus images from 103 eyes from two previously published studies (76 no RDR, 27 RDR).^20^^,^^21^ The first was a study performed at a health care safety-net ophthalmology clinic on 50 adult patients (100 eyes) with diabetes.^21^ Fundus images in this study were captured by a medical resident. The second was a study performed at a quaternary eye care center in Hyderabad that included 52 patients (84 eyes) with a diagnosis of diabetes mellitus or DR.^20^ Images in this study were captured by either a technician with no medical experience, medical student, cornea-trained optometrist, retina-trained optometrist, or vitreoretinal fellow. Images in both studies were acquired using the EyeGo lens attachment (Stanford, CA, USA), an iPhone 5S (1080p and 30Hz video capture; Apple Inc., Cupertino, CA, USA) and a Panretinal 2.2 lens (Volk Optical Inc., Mentor, OH, USA) to capture live video fundus examinations on patients who were already dilated for their clinical examinations. The EyeGo served as the prototype for the device now known as Paxos Scope by DigiSight Technologies (San Francisco, CA, USA).

### Image Set and Preprocessing

**Figure 2.**
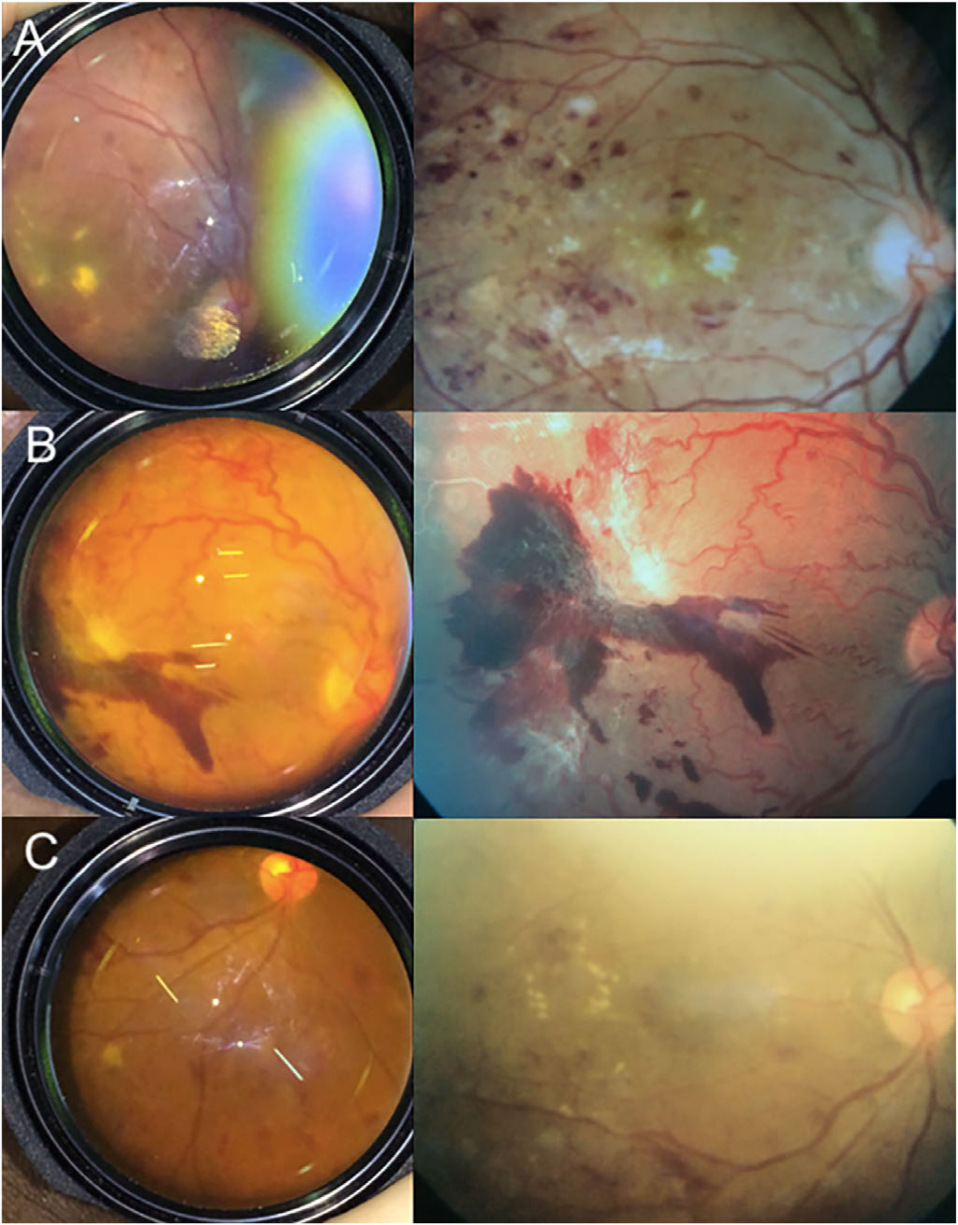
Example mydriatic fundus photographs from the test data set taken from screenshots of live video fundus examinations performed using the EyeGo adapter on an iPhone 5S (Apple Inc.) (*left*) from an FF 450 plus Fundus Camera with VISUPAC Digital Imaging System (Carl Zeiss Meditec Inc., Oberkochen, Germany) (*right*). The deep learning algorithm demonstrated high sensitivity and specificity in spite of glare artifact (A), image warping (B), and lens artifact (C).

**Figure 3.**
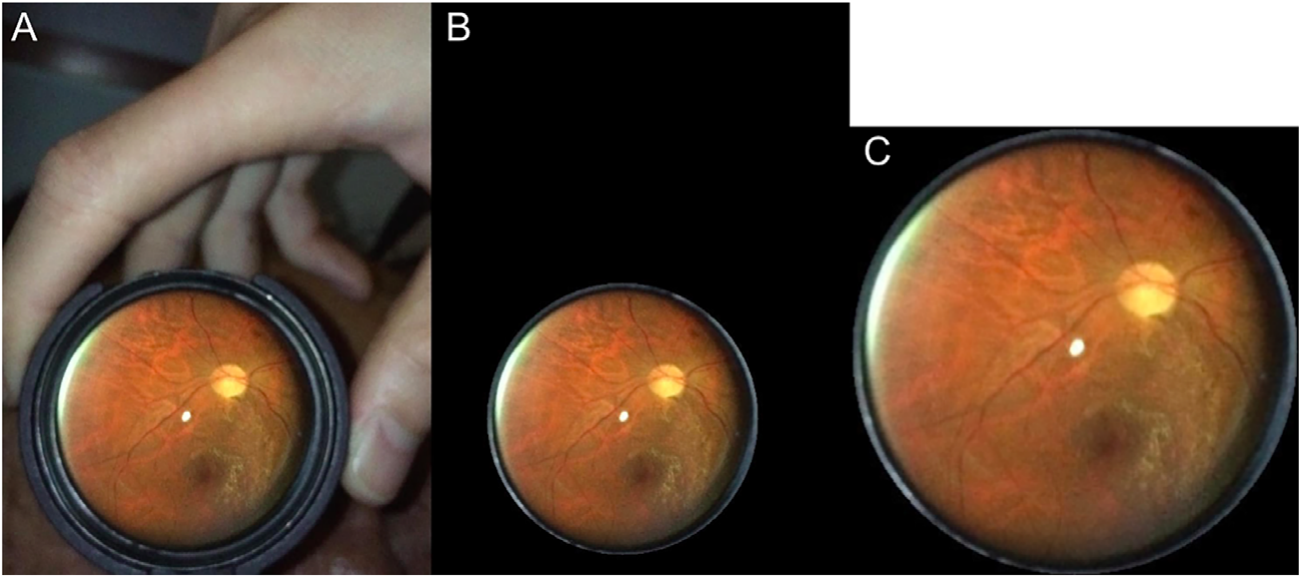
Image preprocessing allowed for standardization of the data set. We used an algorithm on the original image (A) to crop the fundus photo and reduce background noise (B). We then sized the image to a standard resolution of 224 × 224 pixels (C) to match the input size of our chosen model architecture.

To maintain consistency between datasets, allowing for use of an algorithm trained on traditional fundus camera images to be tested on images acquired from a smartphone, all images were preprocessed. To obtain fundus images, we extracted screenshots from live video fundus examinations performed with an SP, the EyeGo adapter, and a Panretinal 2.2 lens (Fig. 2, Fig. 3A). We exported images as screenshots from live video clips filmed at 1080p resolution through the iPhone application Filmic Pro (Cinegenix LLC, Seattle, WA, USA; http://filmicpro.com/) used to provide constant adjusted illumination and video capture in conjunction with the EyeGo. We sized to a standard resolution of 224 × 224 pixels to match the input size of our chosen model architecture. We then used an algorithm to crop the images to reduce background noise in the area captured by the fundus camera or by the SP surrounding the Panretinal 2.2 lens (Fig. 3). This was particularly important in the EyeGo dataset, where the images typically include a hand holding the 20D lens, as well as a partially blocked face.

### Reference Standard

The EyeGo dataset was tested under two different conditions of ground truth. For the first, each eye was graded as having RDR (moderate NPDR or worse) or no RDR based only on the images extracted from live video fundus examinations. This was deemed to be most helpful clinically because the aim of such an algorithm is to determine patients in need of referral to a specialist. Images were graded by two board-certified ophthalmologists with fellowship training in vitreoretinal diseases and surgery. Any disagreement in grading was adjudicated by a third board-certified ophthalmologist with fellowship training in vitreoretinal diseases and surgery. Image grades were then compared to the output of the algorithm, which classified a single representative image chosen for each patient as having RDR or no RDR. For the second, as a sensitivity analysis, 76 eyes of smartphone images from the Hyderabad dataset were graded as having RDR or no RDR based on a grading from the conventional camera fundus photo. Eight eyes were excluded that did not have both conventional fundus photography and EyeGo imaging.

### Statistical Analysis

Python (http://www.python.org) was used to perform DL. To measure the precision-recall trade-off of the algorithm we used area under the receiver operator characteristic curve (AUC), as well as the F-score (scored between 0, worst, and 1, best). Confidence intervals were calculated using 1000 bootstrapped samples of the data.

## Results

We evaluated the performance of our optimal algorithm on the EyeGo smartphone data set and validated its performance on a publicly available data set (Messidor-2) (Table). In contrast with prior studies, our model did not train on any of these data sets prior to validation.^22^^,^^23^ Using the EyeGo smartphone set, our algorithm scored an AUC of 0.89 (95% CI 0.83–0.95) with a sensitivity of 89% (95% CI 81%–100%), specificity of 83% (95% CI 77%–89%), and an F1 score of 0.85 (95% CI 0.80–0.90). Using the Messidor-2 dataset, our algorithm achieved an 87% (95% CI 84%–90%) sensitivity and 80% (95% CI 78%–83%) specificity for detecting RDR with an AUC of 0.92 (95% CI 0.91–0.94) and F1 score of 0.83 (95% CI 0.81–0.85).

**Table.** Average AUC, F-Score, Sensitivity, and Specificity of Nonreferable Diabetic Retinopathy Versus Referable Diabetic Retinopathy Using the EyeGo Smartphone Data Set and a Publicly Available Dataset for Validation

Dataset	No. With RDR	No. Without RDR	AUC (95% CI)	F1 (95% CI)	Sensitivity (95% CI)	Specificity (95% CI)
EyeGo (ground truth EyeGo photo)	27	76	0.89 (0.83–0.95)	0.85 (0.80–0.90)	0.89 (0.81–1.0)	0.83 (0.77–0.89)
EyeGo (ground truth fundus photo)	52	25	0.82 (0.73–0.90)	0.82 (0.75–0.89)	0.83 (0.78–0.91)	0.76 (0.63–0.88)
Messidor-2 (validation)	383	675	0.92 (0.91–0.94)	0.83 (0.81–0.85)	0.87 (0.84–0.90)	0.80 (0.78–0.82)

**Figure 4.**
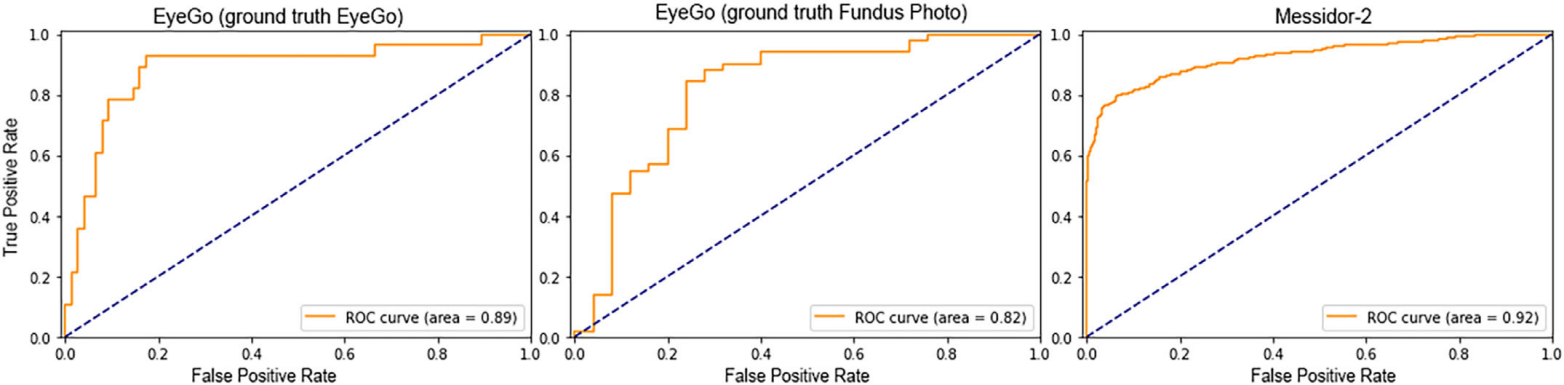
Receiver operating characteristic (ROC) curves for the EyeGo smartphone data set using EyeGo images as ground truth (*left*), EyeGo smartphone data set using Fundus photos as ground truth (*middle*), and Messidor-2 dataset (*right*) demonstrating high reliability of a deep learning algorithm used to screen heterogeneous fundus photos.

Upon sensitivity analysis, where EyeGo images were instead graded for presence of RDR based on traditional fundus photography, the model was able to achieve a sensitivity of 84% (95% CI 78%–91%), specificity of 77% (95% CI 63%–88%), and an AUC of 0.82 (95% CI 0.73–0.90; F1 score 0.83, 95% CI 0.81–0.85) (Fig. 4).

## Discussion

This study demonstrates the feasibility of using a validated DL algorithm to screen patients for RDR using images captured on an SP with a low-cost SP indirect lens adapter. In spite of the presence of multiple artifacts (lens glare, lens particulates/smudging, user hands over the objective lens, Fig. 2) and low-resolution images achieved by users of various levels of medical and ophthalmic training, our model scored a 89% sensitivity and 83% specificity to determine RDR with an AUC of 0.89. This model of automated-AI enabled SP diagnosis provides one possible solution to the problem of screening rising numbers of diabetic patients globally.

Relative to traditional fundus photography, SP are lower cost and more portable and provide wireless access to secure networks for image transmission. Rajalakshmi et al.^24^ previously demonstrated the ability of AI-based DR screening software (EyeArt) to detect DR and sight-threatening DR by fundus photography using Remidio Fundus on Phone, a smartphone-based imaging device that can be used in a handheld mode or fit onto a slit lamp (Remidio Innovative Solutions Pvt. Ltd, Bangalore, India). They graded 296 patients, and their model attained a sensitivity of 96% and specificity of 80% for detecting any DR. Unlike the EyeGo, Remidio devices are difficult to use in handheld mode and are typically used in the slit lamp. This study uniquely demonstrates the feasibility of a remote screener with an SP, lens, and three-dimensional (3D)–printed EyeGo to detect DR.

As expected, we find that the AI model performs best when given smartphone images of the fundus that humans have deemed gradable and correlates well with the human assessment for findings in that field of view. Although the specificity of the model decreases in the “real-world” test where the model was presented with a series of images, many of which are of poor quality and possibly only receiving partial views of the fundus, the performance remained high.

Given the high performance of our model on a public test data set, it is possible that poor image quality by newer users and by nonmedical personnel limited performance. Overall, studies comparing fundus images captured by SPs to slit-lamp biomicroscopy and to images captured using fundus cameras have found considerable agreement, so this modality appears promising.^25^^,^^26^

We did not run the algorithm on the iPhone 5S used to capture the images, but based on our prior analysis, this would be feasible. We previously demonstrated that both iPhone and Android smartphones are capable of running an AI DR algorithm offline for medical imaging diagnostic support.^9^ When tested on an iPhone 5, the real-time runtime performance yielded an average of eight seconds per evaluated image.^9^ Combined with an average recording time with the EyeGo of 73 seconds to obtain a fundus image, nonphysician screeners could provide a diagnosis to patients in less than 1.5 minutes. This would require the development of software to preprocess images incorporated into EyeGo capture, a version of which already exists.

When compared to our model run using photos captured from gold standard fundus photography devices, we achieved a lower AUC, sensitivity, and specificity. In April of 2018 the Food and Drug Administration approved IDx-DR as a breakthrough device for automated diagnosis of diabetic retinopathy.^27^ They did so under an accuracy level of 87.4% sensitivity and 89.5% specificity—a sensitivity below that realized in this study.

It is important to note that the images used in this study were generated from screen captures of video taken at 1080p, using an iPhone 5S that has a much older imaging sensor and camera quality that has been significantly improved in today's smartphones (e.g., iPhone 11). In addition, the EyeGo device used the iPhone's internal light source; the subsequent commercial version of the device (Paxos Scope) uses a variable-intensity, external LED, which enables additional control over illumination and image quality. Currently the commercial Paxos device is configured to capture still images at the modern iPhone full resolution of 12MP 4000 × 3000 dpi—a dramatically higher amount of data than was used in images from this study. Future work is merited to evaluate the image interpretation algorithm developed in this study on images taken with the latest commercial versions of both the camera attachment hardware, as well as smartphone handset.

Additionally, patients required mydriasis for fundus imaging using the EyeGo. Therefore screenings performed outside of the setting of an optometry or ophthalmology clinic would still need to undergo angle evaluation followed by placement of dilation drops. A strength of this study was its use of an algorithm trained with heterogeneous data sets in regard to ethnicity—a feature critical for generalizability first demonstrated by Ting et al.^28^

Overall, we demonstrate the ability of DL to assist in the diagnosis of RDR using low-quality fundus photos attained with an SP, 3D-printed lens attachment, and indirect lens. As it stands, health care workers could bring these portable devices to the homes of individuals unable to travel for screening, dilate, then image the fundus of the patient with a resulting diagnosis within 90 seconds. The efficiency and low cost of this technology will revolutionize the current diagnostic paradigm, allowing for widespread early recognition of DR and prevention of its complications and blindness.
